# Assessing the relevance, efficiency, and sustainability of HIV/AIDS in-service training in Nigeria

**DOI:** 10.1186/1478-4491-12-20

**Published:** 2014-04-17

**Authors:** Randi Burlew, Amanda Puckett, Rebecca Bailey, Margaret Caffrey, Stephanie Brantley

**Affiliations:** 1 CapacityPlus, IntraHealth International Inc, 1776 I Street NW, Suite 650, Washington, DC 20006, USA; 2 Liverpool Associates in Tropical Health, Liverpool, UK

**Keywords:** In-service training, IST, Assessment, Nigeria, PEPFAR, HIV/AIDS, Health systems strengthening

## Abstract

More than three million people in Nigeria are living with HIV/AIDS. In order to reduce the HIV/AIDS burden in Nigeria, the US Government (USG) has dedicated significant resources to combating the epidemic through the President’s Emergency Plan for AIDS Relief (PEPFAR). In-service training (IST) of health workers is one of the most commonly used strategies to improve the quality and coverage of HIV/AIDS services. At USAID/Nigeria’s request, the USAID-funded Capacity*Plus* project conducted an assessment of PEPFAR-funded IST for all cadres of health workers in Nigeria. Using the IST Improvement Framework, developed by the USAID Applying Sciences to Strengthen and Improve Systems Project (ASSIST), as a guide, the authors developed a survey tool to assess the efficiency, effectiveness and sustainability of IST provided between January 2007 and July 2012 by PEPFAR-funded implementing partners in Nigeria. The instrument was adapted to the Nigerian context and refined through a stakeholder engagement process. It was then distributed via an online platform to more than 50 PEPFAR-funded implementing partners who provided IST in Nigeria. A total of 39 implementing partners completed the survey. Our survey found that PEPFAR implementing partners have been providing a wide range of IST to a diverse group of health workers in Nigeria since 2007. Most trainings are developed using national curricula, manuals and/or other standard operating procedures. Many of the partners are conducting Training Needs Assessments to inform the planning, design and development of their training programs. However, the assessment also pointed to a number of recommendations to increase the efficiency, effectiveness and sustainability of PEPFAR-funded IST. These actions are as follows: improve collaboration and coordination among implementing partners; apply a more diverse and cost-effective set of training modalities; allocate funding specifically for the evaluation of the effectiveness of training; improve links between IST and both continuing professional development and pre-service education; require implementing partners to create sustainability plans to transition training from PEPFAR funding to other funding sources; and develop a training information management system to track key aspects of IST, such as the number and types of providers, courses, and participants of PEPFAR-funded IST.

## Background

Nigeria has a population of over 162 million, which is by far the largest number of inhabitants among African countries [1,2]. HIV prevalence among adults in Nigeria is approximately 4.1% [3]. In total, Nigeria has an estimated 3.1 million people living with HIV/AIDS, which accounts for 10% of the global HIV burden [4]. Though HIV prevalence is decreasing, the number of people requiring antiretroviral therapy is on the rise, up from around 850,000 in 2008, to over 1.45 million in 2011 [5]. Health system challenges, including health worker shortages, threaten the progress the Government of Nigeria and development partners have made in combating HIV and AIDS. A sizable number of infected individuals do not get the treatment they need, including the 95% of HIV-positive pregnant women who do not receive prevention of mother-to-child transmission (PMTCT) services, the 73,000 children born infected annually, and the 750,000 individuals in need of antiretroviral therapy who do not receive it [6]. Further, the largest contributor to the global mother-to-child transmission of HIV burden is Nigeria [7].

The United States Government (USG) has dedicated significant resources in Nigeria to combating the HIV/AIDS epidemic through the President’s Emergency Plan for AIDS Relief (PEPFAR). PEPFAR is managed at the country level in Nigeria through the United States Agency for International Development (USAID), the Centers for Disease Control and Prevention, the Department of Defense, and the US Embassy. The majority of PEPFAR activities are conducted through national-level implementing partners that include international nongovernmental organizations and local government agencies [6,8]. Since 2004, PEPFAR has invested more than US$2.5 billion to reduce the HIV/AIDS burden in Nigeria [9]. As a result, the number of sites providing antiretroviral therapy has increased from 24 in 2005 to nearly 400 in 2011, and PEPFAR is currently supporting nearly 1,000 HIV counseling and testing sites in Nigeria [3]. Further, in 2011, PEPFAR reported supporting in Nigeria more than 441,000 individuals on antiretroviral therapy, more than 2.3 million people receiving counseling and testing, and more than 34,000 HIV-positive pregnant women receiving antiretroviral prophylaxis through PMTCT services [10].

A sufficient and qualified health workforce, supported by appropriate training and adequate commodities, is essential for comprehensive HIV/AIDS prevention, care, and treatment. Nigeria, however, is among the 57 countries identified by the World Health Organization as having a human resources for health (HRH) crisis due to insufficient numbers of health providers. Major HRH challenges affecting Nigeria include: shortages of health workers - especially in the north, excess health worker attrition, staff recruitment challenges, poor skills and skills mix among the different cadres of workers, discrepancies in salaries and other conditions of service among states, and a misalignment between pre-service production and training programmes and health priorities [11]. Given the HRH challenges and the urgent need to provide HIV/AIDS prevention, care, and treatment services, more health workers must be educated, trained, and deployed to deliver these services. PEPFAR has worked to harmonize IST for health workers delivering HIV/AIDS services by developing a cadre of master trainers and standardizing training materials [12]. This training is crucial for updating and improving providers’ skills and competencies and represents a large proportion of investments by the Federal Ministry of Health (MOH) and international development partners [6].

In line with PEPFAR priorities for increased ownership and sustainability of IST practices and country systems, more information is needed about how implementing partners collaborate with each other in the management and delivery of HIV/AIDS related IST and to what extent training is effective, efficient, sustainable, and aligned with national priorities. For this reason, USAID/Nigeria requested USAID’s global Capacity*Plus* project to lead a comprehensive effort to assess the implementation of IST by PEPFAR-funded implementing partners with a view to identifying strengths and weaknesses in the implementation of IST to inform future programming.

For the purposes of this study, IST was defined as any PEPFAR-funded HIV/AIDS related training that implementing partners provide for any individual health worker who is engaged in HIV/AIDS related service provision inside or outside of their organization to develop the individual’s skills. This definition of IST is inclusive of all health worker cadres.

## Methods

The assessment aimed to produce a comprehensive analysis of PEPFAR-funded IST in Nigeria, targeted at different cadres of health workers providing HIV/AIDS services at all levels of the health care system and across a wide range of content areas.

The IST Improvement Framework, developed by the Applying Sciences to Strengthen and Improve Systems Project (ASSIST), provided the foundation for developing a survey tool (Additional file 1). The framework was developed through a four-round modified Delphi approach involving a 25-member consensus group comprised of key stakeholder groups and experts, followed by one round of external validation undertaken by 89 individuals representing 26 countries. Stakeholder groups included professional bodies, education and training experts, Ministries of Health, nongovernmental organizations, donors, technical agencies and implementing partners. A targeted literature review summarized evidence for the recommended practices and strategies. The final framework includes 40 recommendations for improving the effectiveness, efficiency, and sustainability of IST, which are grouped into six broad themes: strengthening training institutions and systems; coordination of training; continuum of learning from pre-service to in-service; design and delivery of training; support for learning; and evaluation and improvement of training [13].

A modified version of the stakeholder engagement approach developed by the USAID-funded MEASURE Evaluation project [14] was followed to collect stakeholder feedback on a draft survey tool (Additional file 1). The process developed by MEASURE Evaluation was developed based on work addressing health care and population planning in Africa, Asia, and the Caribbean. The process, as described by MEASURE Evaluation, is not rigid but instead is flexible and offers guidance for engaging stakeholders. Four of the seven steps were used to guide the current project. First, Capacity*Plus*, in collaboration with USAID/Nigeria, identified stakeholder engagement as an important part of the strategy to assess IST. Next, Capacity*Plus* developed a list of stakeholders who are impacted by IST. The list included primary stakeholders (for example, implementing partners, trainees) as well as secondary stakeholders (for example, implementing agencies, relevant government authorities). During the third step, Capacity*Plus* and USAID/Nigeria agreed - due to time constraints as well as the focus on collaboration, overlaps in-service provision, and duplications of effort among implementing partners - to focus on engaging key stakeholders who can significantly influence the process, including implementing partners, development partners, the MOH, and professional councils. Finally, Capacity*Plus* worked to create a stakeholder engagement plan that included meetings with stakeholders in Abuja to provide input into the design of the online assessment tool.

Stakeholders were consulted to identify key issues and challenges, and the most locally-relevant recommendations from the IST Improvement Framework and ensure they were accurately captured in the survey tool (Additional file 1). Key stakeholders included in the process were representatives from 12 PEPFAR-funded implementing partners, three professional councils, four development partners, the human resources for health division of the FMOH, and the National Agency for the Control of AIDS (NACA).

The final survey tool (see attached) included ten general background questions as well as a set of 52 topic-specific questions for each of the following content areas:

• Prevention of Mother to Child Transmission of HIV (PMTCT)

• Male circumcision

• Behavior change

• Counseling and testing

• Infant feeding/nutrition

• Pediatric HIV/AIDS

• Care and treatment

• Orphans and vulnerable children (OVC)

• Laboratory/blood safety

• Supply chain management

• HIV and family planning

• HIV and tuberculosis

• Strategic information (including health information systems, monitoring and evaluation, and surveillance)

• Leadership, policy, financing, or other systems strengthening

In July 2012, the USAID Mission in Nigeria sent an invitation by e-mail to 54 PEPFAR-funded implementing partners in Nigeria, requesting them to participate in the survey delivered via an online platform. Participants were instructed to access the survey platform by clicking a web link in the invitation e-mail. Because the information requested in the survey required referencing many different sources of data and information, the survey was designed so that an incomplete version of responses could be saved and reopened at a later time, and participants were given several weeks to complete the online survey. Three reminder e-mails were sent out to encourage implementing partners to complete the survey. Survey responses were accepted through November of 2012.

Due to technical difficulties with the online platform (for example, due to problems with Internet connectivity and disruptions to the system caused by software upgrades), several implementing partners who completed the survey online were not asked all of the survey questions. In addition, two implementing partners, after experiencing considerable technical difficulties, opted to complete a paper-based version of the survey. In order to ensure the data were of high quality, the authors met with all implementing partners whose surveys were incomplete, contained conflicting responses, or were missing data. Individual meetings were held with 21 implementing partners to address technical issues, gather missing data, and validate responses. In addition, interviews were conducted with eight implementing partners who had not begun the online survey to help them complete the survey.

## Results

A total of 39 PEPFAR-funded implementing partners completed the full IST survey. It is noted that 15 PEPFAR-funded implementing partners did not respond. Therefore, the results of the study may not be generalizable to all PEPFAR-funded IST in Nigeria.

Responses to the survey were analysed according to the following themes:

• Types of courses offered and health workers trained

• Collaboration and coordination between implementing partners

• Variety and appropriateness of curricula and training approaches used

• Application of needs assessments and training evaluations

• Integration of IST content into pre-service education (PSE) and continuous professional development programmes

• Planning for the continued financial support and capacity for IST programmes

• Use of information systems to collect and manage IST data

### Types of courses offered and health workers trained

The number of respondents who reported offering any one training course between January 2007 and July 2012 ranged from one implementing partner providing HIV and family planning and Internal Review Board (IRB) courses to 14 providing counseling and testing and strategic information courses. More than half of the respondents who offered training at any time in each content area also provided this training in 2012. Courses in nine of the fourteen categories were offered in every state and the Federal Capital Territory of Abuja. In addition, training in infant feeding and nutrition was offered in every state except one. The remaining four content areas - behavior change, pediatric HIV/AIDS, leadership, and other/IRB - received less coverage. Table 1 highlights IST training categories and state coverage by implementing partners included in the assessment.

**Table 1:** In-service training (IST) training categories and state coverage by implementing partners

**Training category**	**Total number of IPs who reported providing training in this category since 2007**	**Total number of IPs who reported providing training in this category in 2012**	**Total number of states**^**a **^**in which IPs conducted training since 2007**
PMTCT	12	8	37^a^
Behavior change	12	9	23
Counseling and testing	14	10	37^a^
Infant feeding/nutrition	4	3	36
Pediatric HIV/AIDS	7	4	17
Care and treatment	11	9	37^a^
OVC	13	10	37^a^
Laboratory/blood safety	13	9	37^a^
Supply chain	5	4	37^a^
HIV/family planning	1	1	37^a^
HIV/TB	10	7	37^a^
Strategic information	14	8	37^a^
Leadership, policy, financing	9	7	28
Other	1	1	10

^a^Includes 36 States plus the Federal Capital Territory of Abuja.

OVC, orphans and vulnerable children; PMTCT, prevention of mother to child transmission of HIV.

The respondents reported that training courses targeted trainers, doctors, nurses, midwives, and community health extension workers (CHEWs) in each training category. In addition, they reported training a wide range of ‘other’ health workers which include, but are not limited to, the following: laboratory workers, pharmacists, monitoring and evaluation specialists, government officials, social workers, community volunteers, case managers, nutritionists, and caregivers. Thus, PEPFAR-supported IST is reaching a diverse range of health workers at various levels of the health system. Table 2 outlines the number of implementing partners who reported training a particular cadre of health worker in each training category.

**Table 2:** Health worker cadres by training category

**Training category**	**Trainers**	**Doctors**	**Nurses**	**Midwives**	**Community health extension workers**	**Other**
PMTCT (n = 12)	7	12	12	11	11	11
Behavior change (n = 12)	5	2	3	1	2	5
Counseling and testing (n = 14)	8	9	13	8	11	12
Infant feeding/ nutrition (n = 4)	2	4	4	4	4	1
Pediatric HIV/AIDS (n = 7)	5	6	6	5	3	3
Care and treatment (n = 11)	4	9	10	7	7	7
OVC (n = 13)	6	1	7	3	7	9
Laboratory/blood safety (n = 13)	6	5	6	3	4	11
Supply chain (n = 5)	4	1	2	1	2	5
HIV/family planning (n = 1)	1	1	1	1	1	1
HIV/TB (n = 10)	5	10	8	3	6	7
Strategic information (n = 14)	7	6	7	4	6	12
Leadership, policy, and financing (n = 9)	5	3	2	2	1	7
Other (n = 1)	1	1	1	1	0	1

OVC, orphans and vulnerable children; PTMCT, prevention of mother to child transmission of HIV.

Implementing partners were also asked about the total number of participants trained by cadre. Some of the respondents did not have access to disaggregated figures. In addition, a review of the data revealed a number of inaccuracies. The authors followed up directly with implementing partners to better understand the inaccurate figures. It appears that some implementing partners interpreted the question as referring only to professional or other formal clinical cadres. Thus, the figures they reported did not include the training of government officials or other informal health workers, such as community volunteers and caregivers. Further, some respondents appeared to double count participants. Similar challenges were noted after a closer inspection of the gender disaggregated figures reported by implementing partners. As a result, data about numbers of health workers trained as well as the gender distribution of trained health workers were deemed to be too unreliable to analyse.

### Collaboration and coordination between implementing partners

Implementing partners were asked to describe how they collaborate and coordinate with each other in the planning and delivery of training. In ten of the fourteen content areas, less than half of the respondents reported collaborating with another partner to conduct training (see Table 3). Laboratory/blood safety training had the highest level of reported collaboration. Further context about coordination among IPs was provided during the stakeholder engagement process. Implementing partners that participated in the tool development phase gave isolated reports of contracting with other partners for services, providing training to other partners, and avoiding providing services in locations where other partners with similar focuses may have already begun working. There were also reports of implementing partners working together to harmonize IST curricula and working collaboratively through coordination meetings and technical working groups. The consensus among implementing partners appears to be that coordination is stronger in areas such as PMTCT and blood safety because USAID (through PEPFAR) and other development partners have intervened to encourage or require coordination among IPs.

**Table 3:** In-service training (IST) collaboration among President’s Emergency Plan for AIDS Relief (PEPFAR) implementing partners

**Training category**	**Total number of IPs who reported collaborating with another PEPFAR IP to conduct training in this category**
PMTCT (n = 12)	6
Behavior change (n = 12)	5
Counseling and testing (n = 14)	7
Infant feeding/nutrition (n = 4)	2
Pediatric HIV/AIDS (n = 7)	2
Care and treatment (n = 11)	4
OVC (n = 13)	5
Laboratory/blood safety (n = 13)	10
Supply chain (n = 5)	2
HIV/family planning (n = 1)	0

OVC, orphans and vulnerable children; PTMCT, prevention of mother to child transmission of HIV.

### Variety and appropriateness of curricula and training approaches used

Most respondents reported using national curricula, guidelines, manuals and/or standard training operating procedures to conduct IST (see Table 4). A few implementing partners reported that there was no national curriculum for the specific type of training that they offer (for example, training related to high risk groups). An absence of national curriculum was reported most frequently for the content areas of strategic information, and leadership, policy and financing. In addition, the sole implementing partner that reported providing training in HIV and family planning noted that there was no national curriculum for the particular training they offer. Few implementing partners reported that they use a curriculum that is not based on the national curriculum when such a curriculum is available. It is of note that though a national curriculum may exist for a given content area, it may not cover specific sub-topics addressed in trainings conducted by IPs. This may have contributed to some IPs reporting that a national curriculum exists for a content area in which other IPs report there being no national curriculum.

**Table 4:** Use of national curricula in developing in-service training (IST)

**Training category**	**No national curricula exist**	**Using the national curriculum**	**Using modified version of national curriculum**	**Using curricula that are not based on the national curriculum**
PMTCT (n = 12)	1	8	3	0
Behavior change (n = 12)	2	3	7	0
Counseling and testing (n = 14)	0	12	2	0
Infant feeding/ nutrition (n = 4)	0	4	0	0
Pediatric HIV/AIDS (n = 7)	0	4	2	1
Care and treatment (n = 11)	0	4	7	0
OVC (n = 13)	1	6	6	0
Laboratory/blood safety (n = 13)	2	6	4	1
Supply chain (n = 5)	0	5	0	0
HIV/family planning (n = 1)	1	0	0	0
HIV/TB (n = 10)	0	5	5	0
Strategic information (n = 14)	4	5	5	0
Leadership, policy, financing (n = 9)	4	3	1	1
Other (n = 1)	1	0	0	0

OVC, orphans and vulnerable children; PTMCT, prevention of mother to child transmission of HIV.

The most common training format reported by the implementing partners was face-to-face, group-based training, which requires funding for travel and *per diem* (see Table 5). A number of implementing partners also reported using on-the-job training as a delivery approach. This approach was used by at least one implementing partner in every content area. E-learning was used by very few respondents, and for only half (7) of the content areas. Additional training approaches that were reported in the ‘other’ category were: training of trainers, facility-based step-down training, supportive supervision, mentoring, community-based group training, *practicum*, field visits, and peer mentoring.

**Table 5:** **In-service training** (**IST) training modalities**

**Training category**^**a**^	**Face-to-face with travel and *per diem***	**On the job**	**e-learning**	**Other**
PMTCT (n = 12)	12	9	0	1
Behavior change (n = 9)	9	5	1	3
Counseling and testing (n = 14)	12	6	0	1
Infant feeding/nutrition (n = 4)	4	3	0	0
Pediatric HIV/AIDS (n = 7)	6	6	1	1
Care and treatment (n = 11)	10	7	0	2
OVC (n = 13)	12	7	0	2
Laboratory/blood safety (n = 13)	12	8	0	1
Supply chain (n = 5)	5	1	0	0
HIV/family planning (n = 1)	1	1	1	0
HIV/TB (n = 10)	9	7	2	3
Strategic information (n = 14)	13	8	2	2
Leadership, policy, financing (n = 9)	7	2	1	0
Other (n = 1)	1	0	1	0

^**a**^Participants were able to select more than one answer.

OVC, orphans and vulnerable children; PTMCT, prevention of mother to child transmission of HIV.

### Application of needs assessments and training evaluations

Survey respondents reported using a variety of methods to conduct training needs assessments to inform the planning, design, and development of their IST programmes and to identify participants. Further, there were only a few instances where no training needs assessment was used. Table 6 outlines the prevalence of the various types of needs assessments used by content area. The most frequently reported approaches were pre-training assessments of health workers’ knowledge and skills, and reviewing existing curricula on the subject matter.

**Table 6:** Reported use of training needs assessments

**Training category**^**a**^	**Did not conduct TNA**	**Reviewed health service use/outcome data**	**Reviewed hospital or clinic records**	**Reviewed existing curricula**	**Interviews/ surveys of MOH/licensing staff**	**Interviews/ surveys of beneficiaries**	**Reviewed supervisor/ performance records**	**Observed health worker performance**	**Pre-training assessment of health workers**	**Other**
PMTCT										
(n = 12)	1	6	4	6	4	2	1	5	8	0
Behavior change (n = 12)	2	1	0	4	0	6	0	2	6	3
Counseling and testing (n = 14)	2	3	3	3	2	5	1	5	10	3
Infant feeding/ nutrition										
(n = 4)	0	2	1	3	0	0	1	0	1	1
Pediatric HIV/AIDS (n = 7)	0	1	2	2	0	2	1	1	5	0
Care and treatment (n = 11)	0	4	2	6	2	1	0	6	9	1
OVC (n = 13)	3	2	0	1	2	2	1	1	6	2
Laboratory/blood safety (n = 13)	1	3	4	4	1	0	0	4	7	2
Supply chain (n = 5)	0	2	0	1	1	1	0	0	4	0
HIV/family planning (n = 1)	0	0	1	1	1	0	0	1	1	0
HIV/TB (n = 10)	0	3	2	5	2	2	2	2	6	1
Strategic information (n = 14)	2	1	3	1	1	2	2	2	10	0
Leadership, policy, financing (n = 9)	1	0	0	2	3	1	3	1	2	1
Other (n = 1)	1	0	0	0	0	0	0	0	0	0

^a^Note: survey respondents were able to select more than one answer.

OVC, orphans and vulnerable children; PTMCT, prevention of mother to child transmission of HIV.

The survey included several questions related to the evaluation of training courses. In eight of the training categories, fewer than half of the respondents indicated that they have conducted an assessment or evaluation of the effectiveness or impact of the training (see Table 7). Evaluation appears to happen most frequently for the content areas of PMTCT and laboratory/blood safety. Further, follow up assessments of health worker performance happens more frequently than evaluation of impact. In 11 categories, at least half of the respondents reported having conducted some type of follow up assessment of health workers’ performance. Few implementing partners reported that they made changes to their training curricula based on follow up assessments of health workers. However, those that did make changes reported, among other things, adding modules or modifying existing modules, modifying the course length, improving training slides, including more case studies, improving monitoring and tracking of activities, revising the training modality, and providing physical examples of things like drugs and tools.

**Table 7:** Reported evaluation of in-service training (IST)

**Training category**	**Have you conducted any assessment or evaluation of the effectiveness or impact of this training in Nigeria? (yes)**	**Have you implemented any follow-up assessment that investigated whether/how health worker performance has changed? (yes)**
PMTCT (n = 12)	8	8
Behavior change (n = 12)	3	5
Counseling and testing (n = 14)	6	9
Infant feeding/nutrition		
(n = 4)	1	2
Pediatric HIV/AIDS (n = 7)	3	6
Care and treatment (n = 11)	5	9
OVC (n = 13)	5	7
Laboratory/blood safety (n = 13)	8	9
Supply chain (n = 5)	3	3
HIV/family planning (n = 1)	0	0
HIV/TB (n = 10)	6	5
Strategic information (n = 14)	7	9
Leadership, policy, financing (n = 9)	4	4
Other (n = 1)	1	0

OVC, orphans and vulnerable children; PTMCT, prevention of mother to child transmission of HIV.

### Integration of IST content into pre-service education and continuous professional development programs

Few respondents indicated making any links between their training courses and continuing professional development (CPD) or pre-service education programmes. Though implementing partners frequently offer training participants a certificate or credential upon completion of a course, it is less common for the IST to be recognized by a training institution or university or for IST to qualify for continuing professional development credits from a professional council or lead to promotion or career development within the participants organization/institution (see Table 8). Coordination between implementing partners and professional councils that accredit CPD remain at a very early stage. Content areas that were reported as having been recognized by a training institution or university were: PMTCT; care and treatment; and leadership, policy, and financing. Similarly, content areas for which CPD credits were reportedly offered were: PMTCT; counseling and testing; care and treatment; and leadership, policy, and financing.

**Table 8:** Reported accreditation of in-service training (IST)

**Training category**	**Do graduates receive a certificate or credential?**	**Is the course recognized by a training institution?**	**Do graduates receive CPD credits that are accredited by a professional council?**
PMTCT (n = 12)	11	1	3
Behavior change (n = 12)	10	0	0
Counseling and testing (n = 14)	14	0	1
Infant feeding/nutrition (n = 4)	1	0^a^	0^b^
Pediatric HIV/AIDS (n = 7)	6	0	0
Care and treatment (n = 11)	10	1	1
OVC (n = 13)	9	0	0
Laboratory/blood safety (n = 13)	13	0	0
Supply chain (n = 5)	4	0	0
HIV/family planning (n = 1)	1	0	0
HIV/TB (n = 10)	9	0	0
Strategic information (n = 14)	9	0	0
Leadership, policy, financing (n = 9)	6	2	1
Other (n = 1)	1	0	0

^a^One implementing partner did not answer the question.

^b^One implementing partner did not answer the question.

OVC, orphans and vulnerable children; PTMCT, prevention of mother to child transmission of HIV.

### Planning for the continued financial support and capacity for IST programs

The survey asked implementing partners to describe what they have done to ensure that financial support will be available for the IST they provide to continue providing the training after their PEPFAR funding obligations end. Likewise, respondents were also asked to comment on sustainability plans for human resources and organizational capacity. The majority of respondents reported taking some action to ensure the financial sustainability of their training. However, a number of implementing partners reported that they had not as yet done anything (see Table 9). Funding from the federal government has been secured for two categories of training: laboratory/blood safety, and leadership, policy and financing. State and local government funding has been secured for four categories: PMTCT; counseling and testing; OVC; and laboratory/blood safety.

**Table 9:** Financial sustainability planning for in-service training (IST)

**Training category**	**Nothing yet**	**Federal government funding secured**	**State or local government funding secured**	**Multilateral donor funding secured**	**Bilateral donor funding secured**	**Private donor funding secured**	**Other**
PMTCT							
(n = 12)	3	0	3	1	2	0	3
Behavior change (n = 12)	3	0	0	0	1	1	7
Counseling and testing (n = 14)	5	0	1	0	1	1	6
Infant feeding/ nutrition							
(n = 4)^a^	1	0	0	0	0	1	0
Pediatric HIV/AIDS (n = 7)	5	0	0	0	0	1	1
Care and treatment (n = 11)	6	0	0	0	0	0	5
OVC (n = 13)	5	0	1	1	0	1	5
Laboratory/blood safety (n = 13)	5	2	1	0	0	1	4
Supply chain (n = 5)	2	0	0	0	0	1	2
HIV/family planning (n = 1)	0	0	0	0	0	0	1
HIV/TB (n = 10)	6	0	0	0	0	1	3
Strategic information (n = 14)	8	0	0	1	0	1	4
Leadership, policy, financing (n = 9)	2	1	0	0	0	2	4
Other (n = 1)	1	0	0	0	0	0	0

^a^Two implementing partners did not answer this question.

OVC, orphans and vulnerable children; PTMCT, prevention of mother to child transmission of HIV.

Similarly, although the majority of respondents reported taking some action to ensure that human resources and organizational capacity are available to help sustain training after PEPFAR funding expires, several indicated that they have done nothing yet (see Table 10). It is of note that training in four categories (counseling and testing, OVC, laboratory/blood safety, and strategic information) has been embedded into CPD modules accredited by a professional licensing board, and that some of the training in two categories (supply chain; leadership, policy, and financing) has been incorporated into a pre-service curriculum.

**Table 10:** Sustainability - human resources and organizational capacity

**Training category**	**Nothing yet**	**A public- sector entity**	**A private- sector entity**	**A local partner organization**	**Embedded into a CPD module**	**Embedded into pre-service curricula**	**Other**
PMTCT							
(n = 12)	4	1	0	5	0	0	2
Behavior change (n = 12)	3	3	0	3	0	0	3
Counseling and testing (n = 14)	2	2	0	4	1	0	5
Infant feeding/nutrition							
(n = 4)^a^	0	0	0	2	0	0	0
Pediatric HIV/AIDS (n = 7)	4	0	0	2	0	0	1
Care and treatment (n = 11)	4	1	0	2	0	0	4
OVC (n = 13)	2	2	0	3	2	0	4
Laboratory/blood safety (n = 13)	3	4	2	0	1	0	3
Supply chain (n = 5)	0	0	1	0	0	1	3
HIV/family planning (n = 1)	0	0	0	0	0	0	1
HIV/TB (n = 10)	3	0	1	2	0	0	4
Strategic information (n = 14)	8	1	1	0	1	0	3
Leadership, policy, financing (n = 9)	3	1	1	0	0	1	3
Other (n = 1)	0	1	0	0	0	0	0

^a^Two implementing partners did not answer this question.

OVC, orphans and vulnerable children; PTMCT, prevention of mother to child transmission of HIV.

A number of implementing partners indicated that they are doing ‘other’ things to ensure financial sustainability and continued capacity for training after PEPFAR funding expires. Advocacy to the State Ministry of Health (SMOH) and Federal Ministry of Health (FMOH) for a budgetary allocation was frequently mentioned as well as consistently involving the SMOH and FMOH in training to emphasize the need for continued government funding. Respondents also reported that they are generating financial resources through grants submitted by implementing partners and other community-based organizations, by developing mechanisms to charge fees for courses, by developing relationships with other organizations that can also raise funds for training, and by leveraging other funds. They also reported training government officials, community-based organizations, nongovernmental organizations, master trainers, health workers and facility-based staff to step down training once PEPFAR funding ends. In addition, the responsibility for providing IST has been or will be transitioned to other implementing partners, civil society organizations, training institutions, and other groups that can sustain the training.

### The use of information systems to collect and manage IST data

Most respondents reported using a combination of paper and computer (for example, Excel spreadsheets)-based approaches to capture data on their training courses. However, a few implementing partners reported using only paper-based files, and others reported using more sophisticated, customized databases. For example, one implementing partner reported that their organization uses an automated training database that allows them to track trainees over time. Thirty-one of the 39 respondents said that they are experiencing challenges in managing and using IST data, such as a lack of capacity to collect and analyse data as well as to follow up with trainees after training. The survey also asked partners to consider the potential benefits and challenges of reporting into a standardized training information management system. The respondents noted that in order to be effective the system must be jointly developed between PEPFAR and implementing partners. They perceived that such a system would have several advantages including setting a minimum standard for documenting training, promoting collaboration among implementing partners by highlighting areas of possible joint planning or joint implementation, and reducing duplication of effort. Disadvantages included increasing workloads, the cost associated with training staff on how to use the system, and difficulty customizing data collection (for example, documenting what happens in non-workshop-based training).

## Conclusions and recommendations

PEPFAR implementing partners are already providing IST in Nigeria which is aligned with several recommendations from the IST Improvement Framework. For example, most implementing partners reported aligning their IST with national curricula and standards. The capacity of local trainers is being developed through training of trainers. And, implementing partners are conducting training needs assessment to design and plan courses.

A comparison of the results of the survey in relation to the recommendations of the IST Improvement Framework points to six priority suggestions for improving the efficiency, effectiveness and sustainability of PEPFAR-funded IST in Nigeria. These recommendations are discussed below.

## Increase coordination and collaboration among implementing partners

Coordination and collaboration are particularly important when two or more implementing partners offer training on the same content, target the same cadre, and/or provide training in the same state. Few survey respondents reported that they were collaborating with other PEPFAR-funded implementing partners to plan and conduct IST. However, in many cases, there appeared to be overlap in the work that implementing partners are doing. For example, most implementing partners reported using the national curriculum for their training and within each of the training categories multiple implementing partners reported targeting the same health worker cadres. In addition, for some training categories, multiple partners reported offering the same category of IST within the same state. There is a need to identify opportunities for collaboration between partners in order to use resources more efficiently, reduce duplication of effort, and harmonize training strategies.

## Use a more diverse and cost-effective set of training modalities to decrease the unit cost of training as well as the amount of time workers spend away from their facilities

Face-to-face, group-based training with travel and other related costs is by far the most common approach reported for the delivery of IST. Several implementing partners also reported using on-the-job training as a course delivery modality, and very few reported using e-learning or other self-directed or distance learning approaches. Embracing alternative and innovative training modalities has the potential to reduce costs and to increase the number of health workers that have access to training. On-the-job and distance learning approaches will also help to minimize disruption of services and improve the transfer and application of learning to the workplace.

## Allocate funding specifically for follow up with health workers after training to support the transfer and application of learning and the evaluation of the effectiveness of training

Despite the large investments made in the training of health workers in Nigeria, little is known about the effectiveness of this training. Few survey respondents reported that they are evaluating their training. This is a lost opportunity to assess the impact of the training on health worker performance and service delivery outcomes as well as to provide post-training support and feedback to trainees, to support the transfer and application of learning and identify further training needs. Respondents to the survey noted that one of the biggest obstacles to evaluating training is lack of funding for the costs of such follow up. It is, therefore, suggested to earmark a portion of IST funding for evaluating the effectiveness and impact of the training, including an analysis of the unit cost per trainee. This has the potential to demonstrate the extent to which the training has improved the quality of the HIV/AIDS related services delivered and its impact on the overall HIV/AIDS situation. It will also add to the understanding of best practices in training health workers, to improve the quality and relevance of the training provided, and to increase the effectiveness of PEPFAR-funded training.

## Ensure broader access to new developments in knowledge and technology, as well as sustainability of training, by integrating IST content into pre-service education curricula and continuing professional development (CPD) programs

Many survey respondents reported offering trainees a certificate or credential after completing training courses. However, very few reported that their courses are recognized by a training institution or provide continuing professional development credits recognized by a professional health council. In addition, few implementing partners reported that their IST is embedded in a CPD module or in a pre-service curriculum. Strengthening links between IST and pre-service education as well as between IST and CPD has the potential to promote sustainability, consistency in learning approaches and content between PSE and IST and to improve access to training. Using tutors from local training institutions and building their capacity to train could potentially improve the quality of PSE and IST provided. In addition, being associated with a training institution or health professional council encourages participation, may increase sustainability if trainees are required to pay a nominal fee, raises the visibility of the training, and adds a degree of credibility.

## Create sustainability plans to ensure that financial resources, human resources, and organizational capacity are available to support IST after PEPFAR funding expires

Many survey respondents reported taking steps to ensure that their training is sustained after PEPFAR funding expires. For example, master trainers are being trained in every training category, other funding sources are being secured, and public-sector, private-sector and local organizations are being enabled to take over training. Implementing partners are encouraged to advocate to the government for a line-item budget for HIV/AIDS related IST and strengthening local resources such as trainers. Beyond this, the content and resources developed for the training can be further sustained and strengthened through continued skills transfer, the use of master trainers, and the adaptation and integration of IST content and resources within existing curricula and related training programmes. It is critical to strengthen health training institutions and systems to improve cost-effectiveness, sustainability and accessibility of IST.

## Design an IST information system as a mechanism to support improved planning, coordination, tracking and reporting of PEPFAR-funded IST

Data from the online survey indicate that implementing partners perceive a number of benefits to developing a common IST information system that all implementing partners would report into. However, they note it is important that such a system be designed in collaboration with implementing partners to ensure that it meets their specific needs and to ensure ease of use. Importantly, implementing partners report they believe that a centralized system will promote a more systematic approach to coordination, encourage collaboration, reduce duplication of effort, and potentially increase the effectiveness of training by providing access to national training process and outcome data. As previously discussed, coordination of IST is essential for efficient and effective training. In order to coordinate the planning, execution and follow up of IST, strong data management systems are needed to provide implementing partners with the tools they need.

## Abbreviations

ASSIST: Applying Sciences to Strengthen and Improve Systems Project; CHEW: Community Health Extension Workers; CPD: Continuing Professional Development; FMOH: Federal Ministry of Health; HRH: Human Resources for Health; IRB: Internal Review Board; IST: In-Service Training; MOH: Ministry of Health; NACA: National Agency for the Control of AIDS; OVC: Orphans and Vulnerable Children; PEPFAR: President’s Emergency Plan for AIDS Relief; PMTCT: Prevention of Mother To Child Transmission; PSE: Pre-Service Education; SMOH: State Ministry of Health; TB: Tuberculosis; TNA: Training Needs Assessment; USAID: United States Agency for International Development; USG: United States Government.

## Competing interests

The authors declare that they have no competing interests.

## Authors’ contributions

RB co-led the stakeholder engagement process prior to the study, and led development of the IST assessment tool, analysis of the study results, and the writing of the journal article. AP assisted in the survey tool development and overall management of the activity. AP also contributed to the writing of the journal article and dissemination of the results to IPs in Nigeria. RJB contributed to the development of the stakeholder engagement process and the IST assessment tool. RJB also contributed to the writing of the journal article. MC co-led the stakeholder engagement process, contributed to the development of the IST assessment tool, and provided input to drafts of the journal article. SB managed the online platform for the survey and supported interviews to collect data from IPs who were unable to access the online survey. SB also assisted in analysis of the survey results. All authors read and approved the final manuscript.

## Supplementary Material

Additional file 1 In-Service Training Survey of PEPFAR-funded implementing partners. Click here for file
