# Supplementary material for: Assessing the relevance, efficiency, and sustainability of HIV/AIDS in-service training in Nigeria
Source: Hum Resour Health. 2014 Apr 17;12:20. doi: 10.1186/1478-4491-12-20 (PMC4022275; doi:10.1186/1478-4491-12-20)
Supplement: Additional file 1 — In-Service Training Survey of PEPFAR-funded implementing partners. [file 1478-4491-12-20-S1.doc]

**In-Service Training Survey of PEPFAR-Funded implementing partners**

Thank you for taking the time to complete this survey. Upon the request of USAID Nigeria, the Capacity*Plus* Project is undertaking an assessment of all PEPFAR-funded in-service training (IST) activities in Nigeria. The assessment will include this survey of implementing partners, as well as meetings with implementing partners and other external stakeholders in Abuja. This is not an evaluation of implementing partners but rather an activity designed to provide an overview of how IST is delivered in Nigeria.

For the purposes of this survey, IST refers to the PEPFAR funded training that your organization provides for any individual inside or outside of your organization to develop their skills. The various cadres of health workers that may have benefited from this IST are trainers, doctors, nurses, midwives, community health extension workers (CHEWS), and others, including for example, laboratory workers, pharmacists, monitoring and evaluation officers, HR managers, etc. This training may be delivered in a variety of formats such as off-site, on the job, or online.

This survey asks a ***series of questions about each category of PEPFAR-funded IST*** you have implemented in Nigeria since 2007.

We have generated a ***list of categories of HIV/AIDS IST training*** that are most commonly offered by PEPFAR- funded implementing partners. You will be asked detailed questions about each category of IST you offer so it would be useful for you to have your training records easily accessible as you complete the survey. The categories you will be asked about are:

- Prevention of mother-to-child transmission
- Male circumcision
- Behavior change
- Counseling and testing
- Infant feeding/nutrition
- Pediatric HIV/AIDS
- Care and treatment
- Orphans and vulnerable children (including social welfare workforce)
- Laboratory/blood safety
- Supply chain management
- HIV/family planning
- HIV/tuberculosis
- Strategic information (including health information systems, monitoring and evaluation, and surveillance)
- Leadership, policy, financing or other systems strengthening
- Other

Please try to best fit your IST programs within the categories provided. If one of your trainings fits into two separate categories, include it in the category that covers the major area of emphasis for the training program. If you have implemented a PEPFAR-funded IST training that cannot be placed in any of the categories we have provided, you will be given the opportunity to provide information in the category entitled “**other**.”

You may offer more than one course for any of the IST categories that we have listed above. If that is the case, please think about all of the courses that you offer in that category and select the answer that best fits your entire portfolio.

Please note that any starred (*****) question must be answered to move forward in the survey.

If you need to save your answers and resume filling out the survey at another time, please click the "**Save and Exit**" button at the bottom of the survey. It will provide you with a link to copy and paste into your browser when you want to resume the survey or you can enter your email address and the link will be emailed to you.

Please remember to **adjust the spam settings on your email account** so that the link from the survey comes directly to your inbox and does not end up in your spam folder. The email will be sent from [**rburlew@capacityplus.org**](mailto:rburlew@capacityplus.org)**.**

This ***survey should*** ***only be completed by one person*** at your organization. If more than one person in your organization has received an invitation to complete the survey, please decide amongst yourselves who is the most appropriate person to answer the questions.

1. Name of implementing partner organization and project ______________________________
2. Please provide us with the name of the person completing this survey ____________________
3. Please provide us with the email address of the person completing this survey __________________________________
4. How do you manage your IST data? For example, do you use paper records, an excel spreadsheet, or other means to keep records of your training? Please also tell us what data elements you collect (e.g., gender of participant, date of training, outcome assessment data, etc.). ____________________________________________________________________________________________________________________________________________________________
5. What challenges are you having in managing and using your training information data? ____________________________________________________________________________________________________________________________________________________________
6. If the USG were to create a standardized training information management system (TIMS) that implementing partners were required to report into, what do you foresee as some of the advantages and disadvantages for you as a user of this system? What specific features should the TIMS have to help you use the data to monitor and improve your IST programs?____________________________________________________________________________________________________________________________________________________________
7. Have you conducted any PEPFAR funded IST in the category of prevention of mother- to -child transmission (PMTCT)?
   - No (Skip to question 61)
   - Yes

*Please answer the following questions about the PEPFAR funded IST that you have provided in Nigeria in the category of* ***prevention of mother-to-child transmission*** *since* ***2007****. If you offer more than one training in this category, please think about all of the IST courses you offer in this category and select the answer that best fits your entire portfolio. For example, when you are asked:* ***“How many people have you trained?”*** *we would like to know the total number of people who have completed any of your IST courses in this category.*

1. What is the name of the training course (or courses)? ______________________________________
2. What was the year of first implementation? ______________________________

(Choices: 2007, 2008, 2009, 2010, 2011, 2012)

1. What was the year of the most recent implementation? ____________________

(Choices: 2007, 2008, 2009, 2010, 2011, 2012)

1. How many times have you implemented this type of training since 2007? _____________
2. What is the total number of participants who have completed this training since 2007?

*(Please note: If you have trained individual Trainers and have recorded the total number of additional participants that have received step-down training since 2007—please also include these within the total number of participants who have completed this training.)* __________

1. How many men have completed this training since 2007? __________
2. How many women have completed this training since 2007? ___________

*Please note that the next few questions will ask you about the specific number of health workers you have trained for five (5) separate cadres (trainers, doctors, nurses, midwives and community health extension workers). You will also be asked if you have trained “other” health workers that do not fit into these five cadres (e.g. laboratory workers, pharmacists, monitoring and evaluation officers, human resource managers, etc.). It is possible that a health worker that you have trained fits into more than one category. For example, you may have trained a trainer who is also a doctor. If this is the case, please include that individual in the cadre that best reflects their professional role or job description.* ***Please*** ***do not double-count*** *participants.*

1. Are trainers among the groups of health workers targeted for this training?
   - No (Skip to question 17)
   - Yes
2. How many trainers have completed this training since 2007? _______
3. Are doctors among the groups of health workers targeted for this training?
   - No (Skip to question 19)
   - Yes
4. How many doctors have completed this training since 2007? __________
5. Are nurses among the groups of health workers targeted for this training?
   - No (Skip to question 21)
   - Yes
6. How many nurses have completed this training since 2007? __________
7. Are midwives among the groups of health workers targeted for this training?
   - No (Skip to question 23)
   - Yes
8. How many midwives have completed this training since 2007?________
9. Are community health extension workers (CHEWS) among the groups of health workers targeted for this training?
   - No (Skip to question 25)
   - Yes
10. How many CHEWS have completed this training since 2007? ________
11. Are there any other groups of health workers targeted for this training?
    - No (Skip to question 28)
    - Yes
12. Who are the other groups of health workers targeted for this training? (Please list **ALL** of the other groups of health workers who are targeted for this training. (*For example*, *laboratory workers, pharmacists, monitoring and evaluation officers, human resource managers, etc.*) _______
13. How many of these other health workers have completed this training since 2007? (Please provide a total number that includes all “OTHER” health workers you listed in the previous question.) _______
14. Please list the course objectives. ___________________________________________________________________________________________________________________________________________________________
15. How is the curriculum for this course aligned with the national curricula, guidelines, manuals, and/or standard operating procedures?
    - No national curricula, guidelines, manuals, and/or standard operating procedures for this type of training exists
    - This curriculum is the same as the national curricula, guidelines, manuals, and/or standard operating procedures
    - This is a modified version of the national curricula, guidelines, manuals, and/or standard operating procedures
    - This curriculum is not based on the national curricula, guidelines, manuals, and/or standard operating procedures at all
16. Who developed the curriculum you are using?
    - It was developed by the host country and used without modification (Skip to question 32)
    - It was developed by the host country and used with our modifications (Skip to question 32)
    - It was developed by our organization specifically for our work in Nigeria (Skip to question 32)
    - It was developed by our organization but adapted from work in another country (Skip to question 32)
    - It was developed by another organization for work in Nigeria
    - It was developed by another organization for work in another country
17. What is the name of the organization that developed the curriculum? _____________________
18. Is the version of the curriculum that you are currently using the same as the version you used when you implemented this curriculum the first time or has it been modified?
    - We have always used this version of the curriculum (Skip to question 34)
    - We have modified the curriculum over time
19. How has the curriculum been modified? ________________________________________
20. Please tell us the number of days over which you deliver the curriculum. _____________
21. Please tell us the total number of training hours you use, on average, to deliver training in this category. ____
22. In what format has this course been delivered? (Please check all that apply.)
    - Face-to-face group training requiring travel and per diem
    - On-the-job
    - E-learning
    - Other _____________
23. Did you conduct a training needs assessment before developing and implementing this course? (Please check all that apply.)
    - We did not conduct a training needs assessment
    - We reviewed health service use and outcome data
    - We reviewed existing curricula on the topic
    - We conducted key informant interviews/surveys of MOH and/or national professional licensing organization staff
    - We conducted key informant interviews/surveys of beneficiaries (e.g., community members)
    - We conducted a pre-training assessment of health worker knowledge and skills
    - Other ______________________________
24. How much funding (fully loaded) in US dollars did you receive to implement this training in the most recent fiscal year that you received funding to implement it? ________________
25. For the most recent fiscal year for which you received funding to implement this training, what is the total number of individuals trained or projected to be trained during the fiscal year? _______
26. Do you conduct this training at the national level or at the state level?
    - Only at the national level (Skip to question 42)
    - National and/or state level
27. In what states have you delivered the curriculum? (Please check all that apply.)

| ⃝ Abuja  ⃝ Anambra  ⃝ Enugu  ⃝ Akwa Ibom  ⃝ Adamawa  ⃝ Abia  ⃝ Bauchi  ⃝ Bayelsa  ⃝ Benue | ⃝ Borno  ⃝ Cross River  ⃝ Delta  ⃝ Ebonyi  ⃝ Edo  ⃝ Ekiti  ⃝ Gombe  ⃝ Imo  ⃝ Jigawa | ⃝ Kaduna  ⃝ Kano  ⃝ Katsina  ⃝ Kebbi  ⃝ Kogi  ⃝ Kwara  ⃝ Lagos  ⃝ Nasarawa  ⃝ Niger | ⃝ Ogun  ⃝ Ondo  ⃝ Osun  ⃝ Oyo  ⃝ Plateau  ⃝ Rivers  ⃝ Sokoto  ⃝ Taraba  ⃝ Yobe  ⃝ Zamfara |
| --- | --- | --- | --- |

1. In terms of sustainability, what have you done to ensure there is financial support for this training beyond when your funding ends? (If more than one answer applies, please choose the option that reflects the major source of financial support). Only one answer is allowed.
   - Nothing yet (Skip to question 44)
   - Federal government funding has been secured to continue this training once our funding ends (Skip to question 44)
   - State or local government (LGA) funding has been secured to continue this training once our funding ends (Skip to question 44)
   - Other donor funding *(multilateral)* has been secured to continue this training once our funding ends
   - Other donor funding *(bilateral)* has been secured to continue this training once our funding ends
   - Other donor funding *(private)* has been secured to continue this training once our funding ends
   - Other _______________________________ (Skip to question 44)
2. Please tell us the name of the donor that is providing funding to sustain this training when your PEPFAR funding ends. ______________________________________
3. In terms of sustainability, what have you done to ensure there are human resources and/or organizational capacity to continue this training beyond when your PEPFAR funding ends?
   - Nothing yet (Skip to question 46)
   - A public sector entity has been identified and enabled to provide the human resources needed to continue this training once our funding ends
   - A private sector entity has been identified and enabled to provide the human resources needed to continue this training once our funding ends
   - A local partner organization has been identified enabled to provide the human resources needed to continue this training once our funding ends
   - This training has been embedded into a continuing professional development module accredited by a professional licensing board
   - This training has been incorporated into pre-service curricula
   - Other ______________________________________________ (Skip to question 46)
4. Please tell us the name of the entity that will provide the human resources needed to sustain this training once your PEPFAR funding ends. __________________________
5. Since receiving PEPFAR funding to implement this course, have you collaborated with any other PEPFAR implementing partners or organizations doing similar work to implement this training?
   - No (Skip to question 48)
   - Yes- A PEPFAR-funded implementing partner
   - Yes- Another organization that is not a PEPFAR-funded implementing partner
   - Not sure if the collaborating organization is PEPFAR funded or not
6. Please tell us the name of the organization that you have collaborated with to implement this training. _________________________________________________
7. Have you conducted any assessment or evaluation of the impact or effectiveness of this training (beyond counting number of people trained) in Nigeria that involves collecting data from or about your trainees?
   - No (Skip to question 52)
   - Yes
8. At what points in time do you collect data from or about your trainees? (Please check all that apply.)
   - Immediately before training (Skip to question 51)
   - Immediately after training (Skip to question 51)
   - After some time has passed since the training
9. How long, in months, after training do you collect data from or about your trainees? ________
10. What methods do you use to collect data from or about your trainees? (Please check all that apply.)
    - Interviews
    - Focus group discussions
    - Surveys
    - Skills assessments
    - Health sector reviews
    - Supportive supervision
    - Other ____________________________________
11. Have you implemented any follow-up assessment that investigated whether/ how health worker performance has changed as a result of the training course provided?
    - No (Skip to question 56)
    - Yes
12. Please describe the methods you used to conduct your follow up assessments that explicitly investigated whether/ how health worker performance has changed as a result of the training course provided. ____________________________________________________________________________________________________________________________________________________________
13. Have you made any changes to the curriculum based on the results of your follow up assessments that explicitly investigated whether/ how health worker performance has changed as a result of the training course provided?
    - No (Skip to question 56)
    - Yes
14. Please describe the changes that you made to your curriculum based on the results of your follow up assessments on learning outcomes and performance. ____________________________________________________________________________________________________________________________________________________________
15. Do you offer trainees a certificate or credential for successfully completing this course?
    - No
    - Yes
16. Is this training accredited by a training institution or university?
    - No (Skip to question 59)
    - Yes
17. What is the name of the training institution or university that accredits the training? ____________________________________________________________________
18. Do participants who complete this course receive continuing professional development credits that are accredited by a professional council?
    - No (Skip to question 61)
    - Yes
19. Which professional councils accept the continuing professional development credits participants receive for taking this course? (Please check all that apply.)
    - Nursing and Midwifery Council of Nigeria
    - Medical and Dental Council of Nigeria
    - Pharmacists Council of Nigeria
    - Medical Laboratory Science Council of Nigeria
    - Community Health Practitioners Registration Board of Nigeria
    - Other ________________________

*Thank you for answering questions about the PEPFAR funded IST that you conduct in Nigeria in the category of prevention of mother-to-child transmission.*

1. Do you conduct any PEPFAR funded HIV/AIDS IST in the category of male circumcision?
   - No (Skip to question 115)
   - Yes

***Repeat questions 8-61 for each category in which you conduct training.***

1. What is your organization’s biggest challenge in ensuring the effectiveness of your IST?
2. What is your organization’s biggest challenge in ensuring the efficiency of your IST?
3. What is your organization’s biggest challenge in sustaining your PEPFAR funded IST?

*This is the end of the survey. Thank you so much for your participation. Your answers will help us to better understand how IST is planned, implemented, and evaluated in Nigeria. As a final step in this assessment, we would like to ask you to email us copies of the curricula for the PEPFAR funded IST courses that you referenced while completing the survey. Please email the curricula as well as any questions to Randi Burlew at* [*rburlew@capacityplus.org*](mailto:rburlew@capacityplus.org)*.*
